# Human CD4^+^ T cells require exogenous cystine for glutathione and DNA synthesis

**DOI:** 10.18632/oncotarget.5213

**Published:** 2015-09-03

**Authors:** Trine B. Levring, Martin Kongsbak, Anna K. O. Rode, Anders Woetmann, Niels Ødum, Charlotte Menné Bonefeld, Carsten Geisler

**Affiliations:** ^1^ Department of Immunology and Microbiology, Faculty of Health and Medical Sciences, University of Copenhagen, Copenhagen, Denmark

**Keywords:** Immunology and Microbiology Section, Immune response, Immunity, T cell, cystine, glutathione, ribonucleotide reductase, DNA synthesis

## Abstract

Adaptive immune responses require activation and expansion of antigen-specific T cells. Whereas early T cell activation is independent of exogenous cystine (Cys_2_), T cell proliferation is dependent of Cys_2_. However, the exact roles of Cys_2_ in T cell proliferation still need to be determined. The aim of this study was to elucidate why activated human T cells require exogenous Cys_2_ in order to proliferate. We activated purified naïve human CD4^+^ T cells and found that glutathione (GSH) levels and DNA synthesis were dependent on Cys_2_ and increased in parallel with increasing concentrations of Cys_2_. Vice-versa, the GSH synthesis inhibitor L-buthionine-sulfoximine (BSO) and inhibition of Cys_2_ uptake with glutamate inhibited GSH and DNA synthesis in parallel. We further found that thioredoxin (Trx) can partly substitute for GSH during DNA synthesis. Finally, we show that GSH or Trx is required for the activity of ribonucleotide reductase (RNR), the enzyme responsible for generation of the deoxyribonucleotide DNA building blocks. In conclusion, we show that activated human T cells require exogenous Cys_2_ to proliferate and that this is partly explained by the fact that Cys_2_ is required for production of GSH, which in turn is required for optimal RNR-mediated deoxyribonucleotide synthesis and DNA replication.

## INTRODUCTION

A key point in specific immune responses is the activation and proliferation of antigen-specific T cells. We and others have shown that exogenous cystine (Cys_2_), the oxidized dimeric form of cysteine (Cys), is dispensable for early T cell activation but indispensable for T cell proliferation [1, 2]. Due to the oxidizing environment, approximately 90% of Cys is found as Cys_2_ in the extracellular space. Plasma thus contains 50–100 μM Cys_2_, whereas the concentration of Cys is very low compared to other amino acids [3, 4]. Cys is transported over the cell membrane predominantly by the neutral amino acid transporters ASCT1 (SLC1A4) and ASCT2 (SLC1A5), whereas Cys_2_ exclusively is transported by the x_c_^−^ cystine/glutamate antiporter composed of the heavy subunit 4F2 hc (CD98) and the light subunit xCT (SLC7A11) [5, 6]. Inside the cell, Cys_2_ is rapidly reduced to Cys due to the reducing intracellular milieu [7]. Naïve T cells express no or very low levels of x_c_^−^ and are deficient in transportation of Cys_2_ over the plasma membrane [2, 8, 9], but shortly after activation x_c_^−^ becomes strongly upregulated in both human and mouse T cells and provide the cells with the required amount of Cys_2_/Cys needed for proliferation [1, 8, 10]. However, the exact mechanisms that are dependent on Cys in order for T cells to proliferate still need to be identified.

Cell proliferation requires doubling of the cell constituents including replication of the DNA. Availability of the deoxyribonucleoside triphosphates (dNTP) DNA building blocks is the limiting factor in DNA synthesis. In resting cells, the dNTP pools are kept low, but during DNA synthesis in the S phase the dNTP pool increases several fold [11–14]. Insufficient levels of dNTP results in an elongation of the S phase [15]. Furthermore, it is essential that the dNTP levels are neither too high nor unbalanced as dysregulated dNTP levels lead to an increased rate of mutagenesis [16]. Thus, DNA synthesis requires a strictly regulated continuous *de novo* synthesis of dNTPs. Ribonucleotide reductase (RNR) is a key enzyme for dNTP generation. RNR generates deoxyribonucleoside diphosphates (dNDP) through reduction of the corresponding ribonucleoside diphospate (NDP) [11–14]. After conversion from NDP, dNDP is finally phosphorylated to dNTP. RNR is responsible for maintaining the total dNTP pool size and ensuring that the levels of the four dNTPs are balanced. During the catalysis, the 2′-OH group of the NDP ribose ring is reduced to hydrogen. In this process, a disulfide bridge is generated in the active site of RNR [11–14]. In order for RNR to restore its original configuration and be capable of catalyzing a new round of NDP reduction, external thiol-dependent systems are required to reduce the disulfide bridge in the active site. Thioredoxin (Trx) and later glutaredoxin (Grx) were discovered as thiol electron donors for RNR in *E. coli* [17, 18]. Unlike Trx, Grx was found to be functional as an electron donor only in the presence of glutathione (GSH). In *E. coli*, the glutaredoxin-glutathione (Grx/GSH) system is the most efficient electron donor for RNR [12, 19], but in yeast the major electron donor is Trx [20]. Which system that is functional in mammalian cells is less clear, although cell-free studies using recombinant mouse RNR have indicated that both Trx and the Grx/GSH system can reduce RNR [21].

GSH is synthesized in the cytosol and consists of the amino acids glutamate, Cys and glycine, where Cys is believed to be the limiting amino acid [22, 23]. Several studies have shown that GSH plays essential roles in T cell function and proliferation [2, 24–29]. These studies indicate that GSH is not required during the early steps of T cell activation, but that it is essential for processes close to or at the level of DNA synthesis. However, the exact role(s) of GSH during T cell proliferation still needs to be determined. Taking into account that Cys is required for GSH synthesis [22, 23], it could be speculated that in order to proliferate, human T cells need exogenous Cys_2_ to generate sufficient amounts of GSH that subsequently supply the reducing power required for RNR activity and thereby dNTP synthesis and DNA replication. The aim of this study was to identify processes in which exogenous Cys_2_ is required in order for T cell proliferation to take place.

## RESULTS

### Exogenous cystine is required for GSH and DNA synthesis

**Figure 1:**
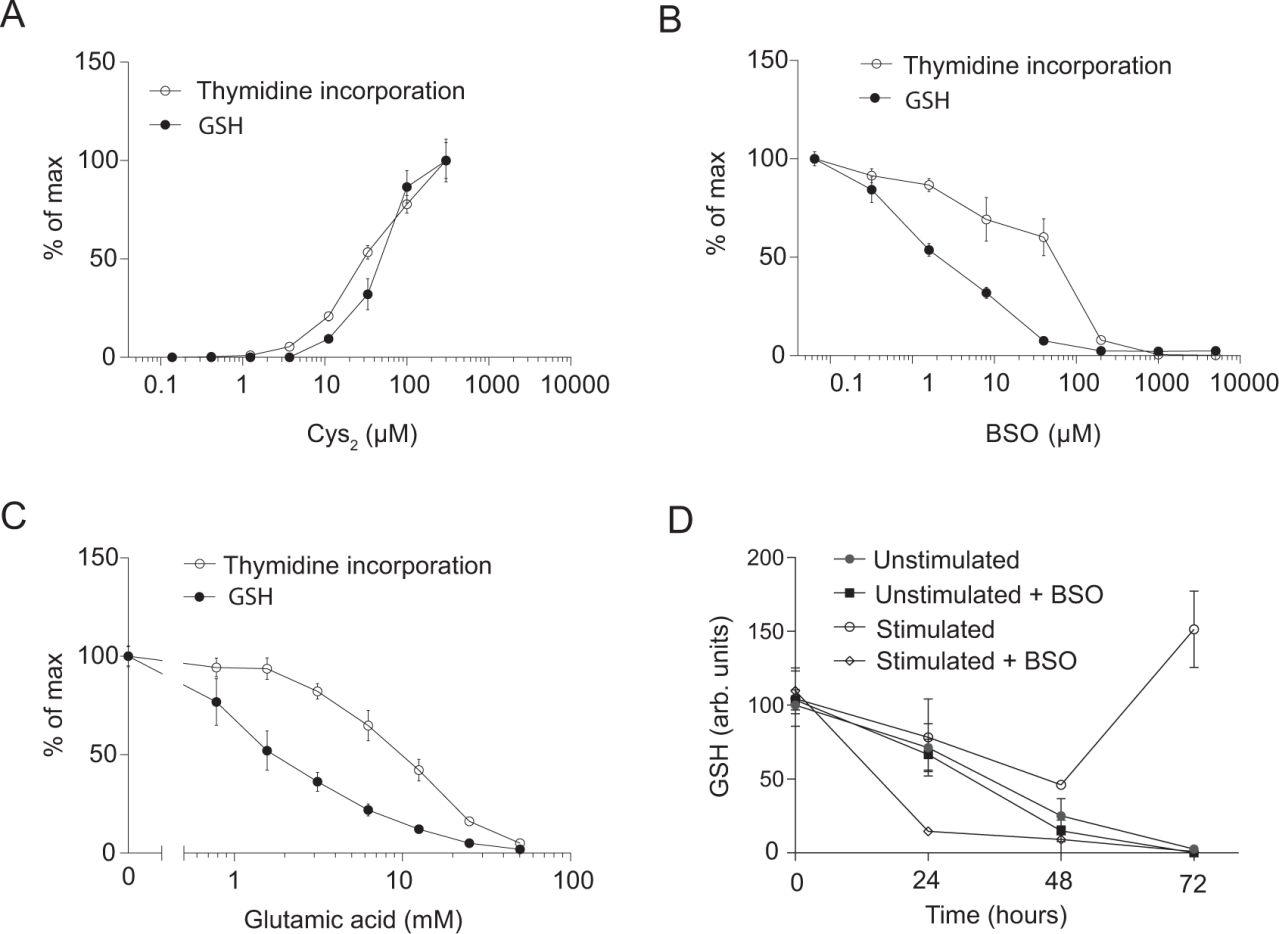
Exogenous cystine is required for GSH and DNA synthesis **A.** Thymidine incorporation and GSH levels of CD4^+^ T cells activated for 3 days in Cys_2_-free DMEM medium supplemented with increasing amounts of Cys_2_. **B.** Thymidine incorporation and GSH levels of CD4^+^ T cells activated for 3 days in X-VIVO 15 medium supplemented with increasing levels of BSO. **C.** Thymidine incorporation and GSH levels of CD4^+^ T cells activated for 3 days in DMEM medium supplemented with the indicated concentrations of glutamic acid. (A–C) Data are shown as percentage of maximum ^3^H-thymidine incorporation and GSH levels, respectively. **D.** GSH levels of CD4^+^ T cells either left unstimulated or activated for 0–72 hours in the presence or absence of 100 μM BSO. (A–D) Data are given as mean ± SEM from at least two experiments, each carried out in triplicates.

We have recently shown that activation of human T cells as assessed by the upregulation of the activation markers CD25 and CD69 as well as IL-2 production is independent of exogenous Cys_2_. In contrast, DNA synthesis as measured by ^3^H-thymidine incorporation is strictly dependent on the presence of Cys_2_ in the tissue culture medium [1]. As Cys is supposed to be the limiting amino acid in the synthesis of GSH [22, 23] and it has been suggested that GSH can provide the reducing power necessary for RNR to produce dNDP required for dNTP and DNA synthesis [12], we first wanted to study whether a correlation exists between the requirement for Cys and GSH and DNA synthesis in T cells. To study a homogenous T cell population, we purified naïve CD4^+^ T cells from buffy coats obtained from healthy blood donors. The resulting cell population consisted of approximately 98% CD4^+^CD45RA^+^CD25^−^CD69^−^ T cells [1]. We stimulated the purified T cells with anti-CD3 and anti-CD28-coated Dynabeads for 3 days in Cys_2_-free DMEM tissue culture medium supplemented with increasing concentrations of Cys_2_. We found that both GSH levels and DNA synthesis were strictly dependent on Cys_2_ (Figure 1A). The levels of GSH and DNA synthesis increased in parallel with increasing concentrations of Cys_2_ in the medium. To determine whether the dependency of Cys_2_ for DNA synthesis was due to the requirement of Cys_2_ for GSH synthesis or for other GSH-independent mechanisms, we next stimulated the cells for 3 days in X-VIVO 15 tissue culture medium that contains 290 μM Cys_2_ with various concentrations of BSO that specifically inhibits synthesis of GSH. BSO concentrations of 100 μM and above completely depleted GSH in the T cells. Likewise, DNA synthesis was impaired by BSO, although we noticed a small displacement between the two curves (Figure 1B). These results suggested that DNA synthesis is dependent on Cys_2_ due to the requirement of Cys_2_ for GSH synthesis and not for other mechanisms. To further investigate the requirement of exogenous Cys_2_ for GSH and DNA synthesis, we inhibited the uptake of Cys_2_ by incubation of the cells with increasing concentrations of glutamic acid. Glutamic acid is the main exchange substrate in the x_c_^−^ cystine/glutamate antiporter-mediated uptake of Cys_2_ and is a highly specific inhibitor of Cys_2_ uptake [6]. We found that glutamic acid inhibited GSH and DNA synthesis in parallel (Figure 1C). As for BSO-treated cells, we noticed a small displacement between the curves for GSH and DNA synthesis suggesting that some residual DNA synthesis can take place in the absence of GSH. From these experiments we could conclude that T cells require exogenous Cys_2_ to produce GSH, and that they need GSH for normal DNA synthesis to take place.

To further study GSH occurrence and production, we isolated naïve CD4^+^ T cells and left them either unstimulated or stimulated them with anti-CD3/CD28 beads in X-VIVO 15 tissue culture medium with or without 100 μM BSO for 0 to 72 hours. We found that freshly purified naïve CD4^+^ T cells contain GSH, and that the GSH level slowly declines in unstimulated T cells independently of the presence of BSO (Figure 1D). This indicated that naïve unstimulated T cells consume or lose GSH to the tissue culture medium and that they do not actively produce GSH *in vitro*. Stimulation of the T cells in the presence of BSO lead to a rapid decline in GSH indicating that T cell stimulation accelerates the consumption or loss of GSH. T cell stimulation in the absence of BSO initially led to a decline in GSH concentration, but after 72 hours the GSH concentration exceeded the initial GSH concentration in naïve T cells (Figure 1D), indicating that *de novo* synthesis of GSH takes place in activated CD4^+^ T cells.

### Thioredoxin and GSH can partly substitute for each other in DNA synthesis

**Figure 2:**
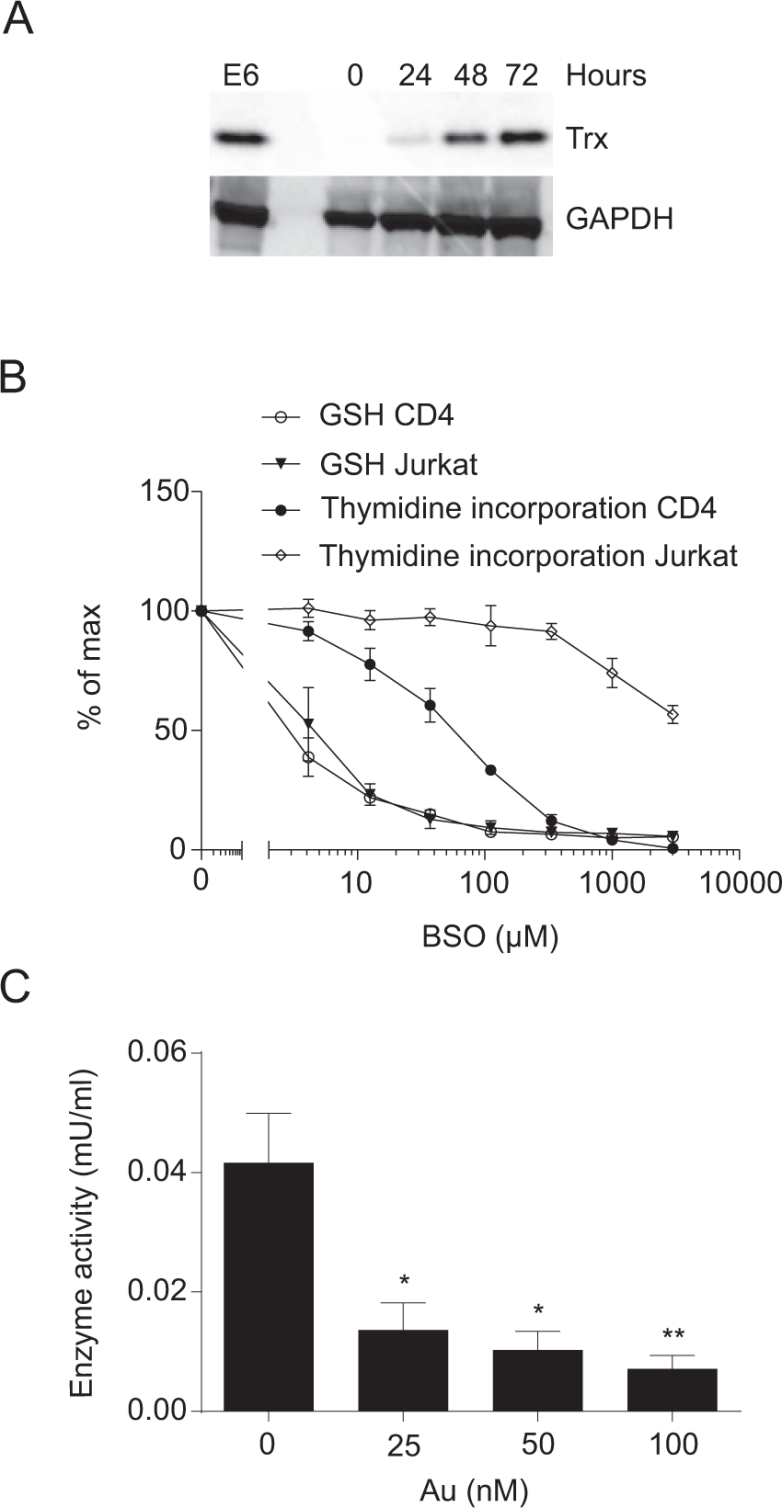
Thioredoxin in primary CD4^+^ T cells and Jurkat cells **A.** Representative Western blot of Trx and GAPDH (loading control) in Jurkat cells (E6) and CD4^+^ T cells activated for 0–72 hours in X-VIVO 15 medium. **B.** Thymidine incorporation and GSH levels of CD4^+^ T cells activated in X-VIVO 15 medium and Jurkat cells cultured in RPMI-1640 medium for 48 hours in the presence of the indicated concentrations of BSO. Data show mean ± SEM of two experiments carried out in duplicates. **C.** Thioredoxin reductase activity of CD4^+^ T cells activated for 3 days in X-VIVO 15 medium with the indicated concentrations of Au. Data show mean ± SEM of four experiments.

From the experiments shown in Figure 1A–1C we could conclude that exogenous Cys_2_ is required for GSH production and that GSH is required for optimal DNA synthesis in activated CD4^+^ T cells. However, we also noted that some residual DNA synthesis took place even in cells completely depleted of GSH (Figure 1B and 1C). This indicated that GSH can be replaced by other reducing agents during DNA synthesis. It has been suggested that Trx and the Grx/GSH system can substitute for each other in providing the reducing power required for DNA synthesis [12, 21], and we wanted to see whether this could also be the case in human T cells. We therefore determined the expression of Trx in human CD4^+^ T cells stimulated for 0 to 72 h and compared it with Trx expression in the human leukemic T cell line Jurkat. We found that naïve CD4^+^ T cells express very low levels of Trx and that T cell stimulation induces significant Trx upregulation. Following 72 h of stimulation, primary T cells expressed Trx levels similar to those of Jurkat cells (Figure 2A).

If Trx can substitute for GSH, it would be expected that DNA synthesis in Jurkat cells with a constitutive high level of Trx would be more resistant to GSH depletion than primary T cells. We consequently treated primary T cells and Jurkat cells in parallel with increasing concentrations of BSO for 48 h and subsequently measured GSH levels and DNA synthesis. We found that even though GSH levels decreased at equal rates in the two cell types in response BSO treatment, DNA synthesis in Jurkat cells was much more resistant to BSO treatment than DNA synthesis in primary T cells (Figure 2B). This supported that Trx can substitute for GSH in DNA synthesis in human T cells.

Trx-mediated reduction of other proteins results in oxidation of Trx [30, 31]. In order for Trx to be capable of catalyzing a new round of reduction, Trx must it-self be reduced. The Trx reductases (TrxR) are the only enzymes known to reduce oxidized Trx, and inhibition of TrxR impairs the redox function of Trx [30–32]. Auranofin (Au) is an irreversible inhibitor of TxrR [33]. To determine the concentration of Au required to inhibit TrxR in primary T cells, we stimulated naïve CD4^+^ T cells with CD3/CD28 beads for 3 days in the presence of increasing concentrations of Au and subsequently measured the TxrR activity. We found that Au already at 25 nM significantly inhibited TrxR activity (Figure 2C).

**Figure 3:**
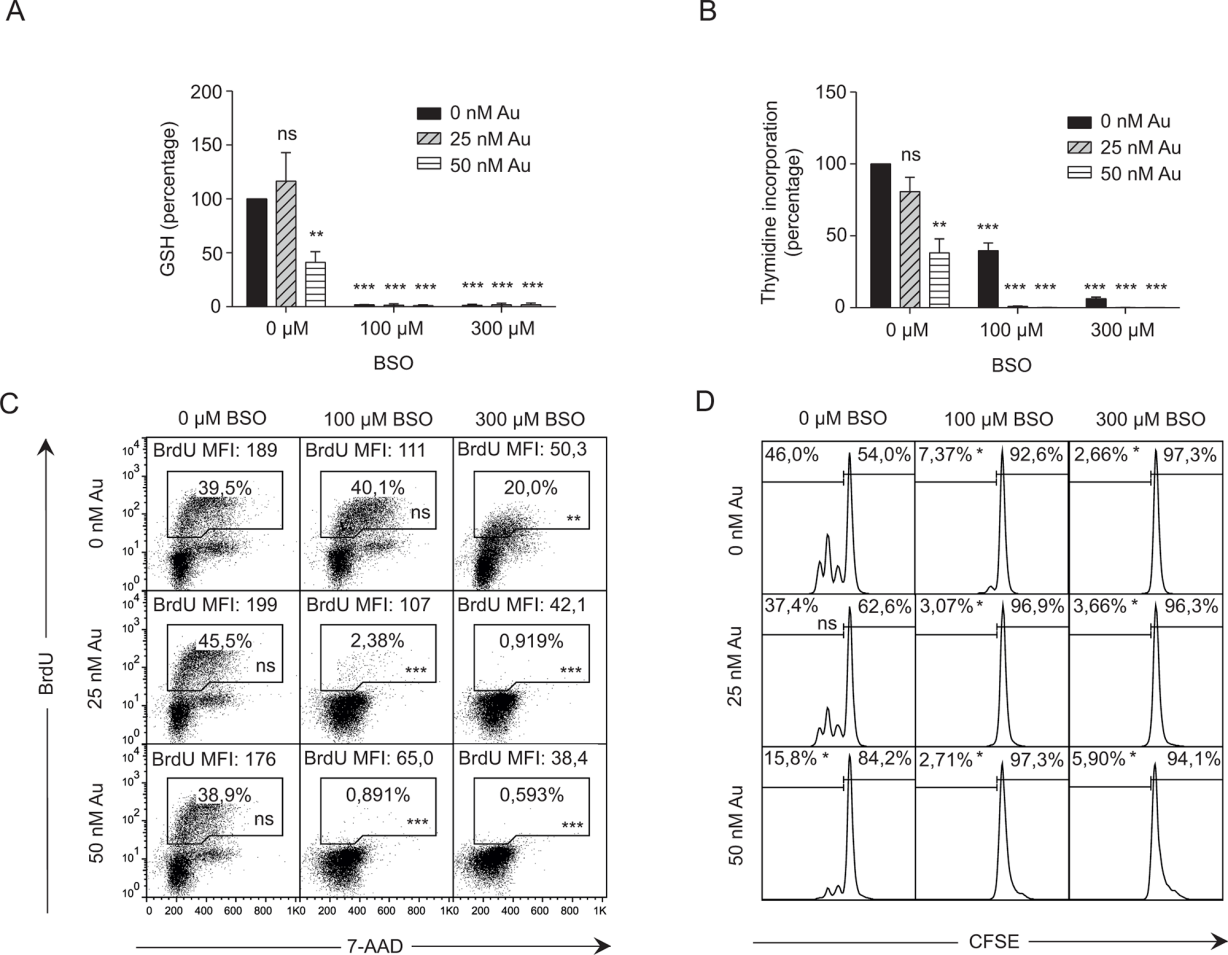
Thioredoxin and GSH can partly substitute for each other in DNA synthesis **A.** GSH levels and **B.** thymidine incorporation of CD4^+^ T cells activated for 3 days in X-VIVO 15 medium in the presence of the indicated concentrations of BSO and Au. Data show mean ± SEM of three experiments carried out in duplicates. **C.** DNA synthesis as measured by BrdU incorporation and 7-AAD staining and **D.** cell division as measured by CFSE dilution of CD4^+^ T cells activated for 3 days in X-VIVO 15 medium in the presence of the indicated concentrations of BSO and Au. Data show representative results from two experiments carried out in duplicates.

To further investigate whether GSH and Trx can substitute for each other during DNA synthesis in primary T cells, we next stimulated CD4^+^ T cells in a checkerboard titration of BSO and Au for 3 days. We subsequently measured GSH levels, DNA synthesis as ^3^H-thymidine and BrdU incorporation, and T cell division as dilution of CFSE. We found that the low dose of Au (25 nM) did not significantly affect DNA synthesis or cell division in the absence of BSO (Figure 3A–3D). Treatment with the low dose of BSO (100 μM) without Au completely abrogated GSH expression and reduced ^3^H-thymidine and BrdU incorporation and cell division, although it did not affect the percentage but only the mean fluorescence intensity (MFI) of cells that incorporated BrdU (Figure 3A–3D). Interestingly, the combination of the low dose of Au (25 nM) and BSO (100 μM) completely abolished ^3^H-thymidine and BrdU incorporation and cell division (Figure 3B–3D). These experiments demonstrated that inhibition of either GSH or Trx still allows some DNA synthesis, whereas simultaneous inhibition of GSH and Trx completely abolishes DNA synthesis. This further supported that GSH and Trx can partly substitute for each other during DNA synthesis.

### GSH and/or Trx is required for dNTP production

**Figure 4:**
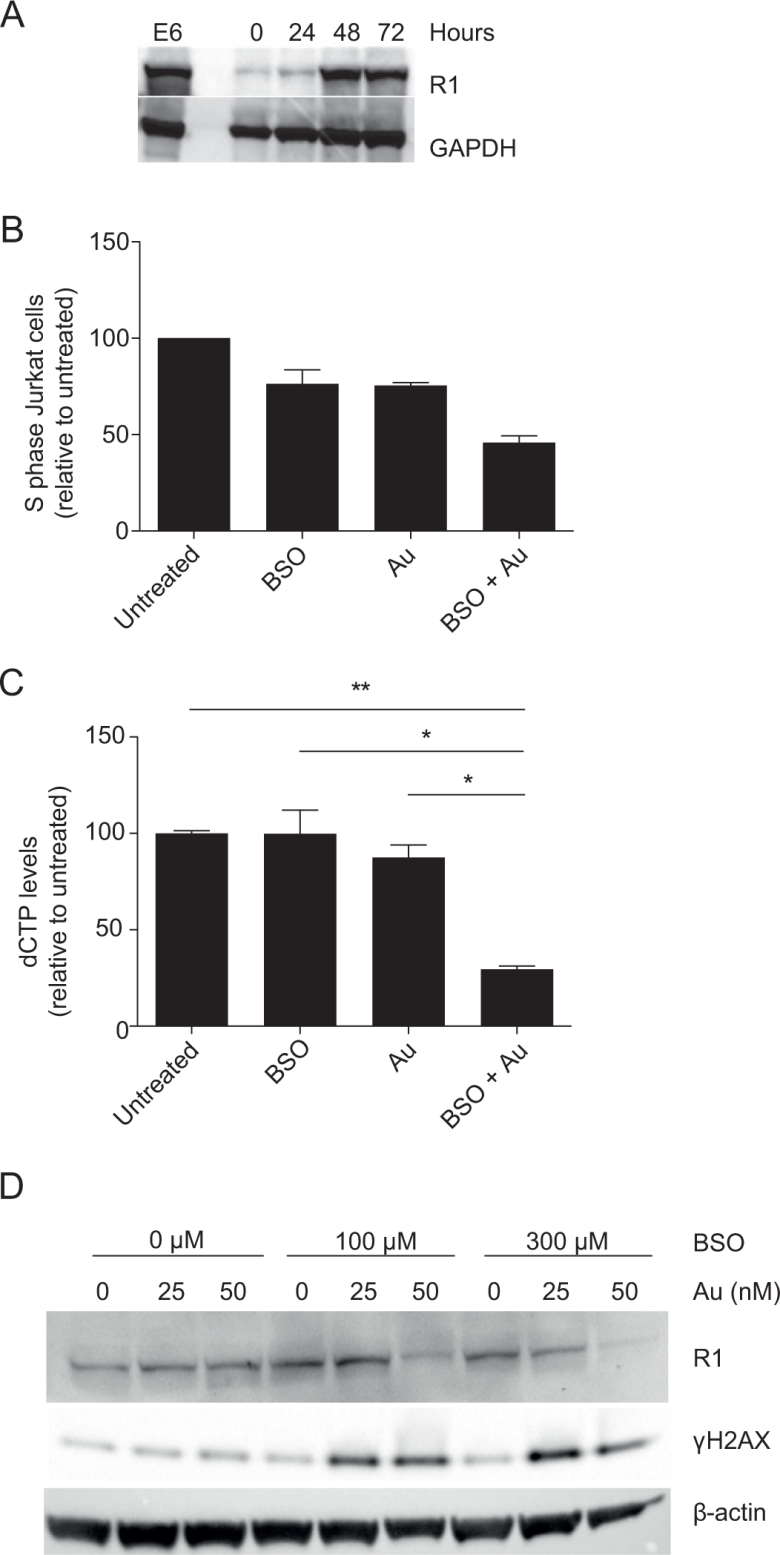
Inhibition of GSH and Trx impairs dNTP production and induces γH2AX **A.** Representative Western blot of R1 and GAPDH (loading control) in Jurkat cells and CD4^+^ T cells activated for 0–72 hours. The same gel as shown in Figure 2A was stripped and reblotted with antibodies against R1. **B.** The fraction of Jurkat cells in S phase relative to untreated cells 16 hours after release from serum starvation. The cells were either left untreated, treated with 300 μM BSO, 200 nM Au or a combination of both. **C.** dCTP levels in cell cycle-synchronized Jurkat cells treated as in (B) dCTP measurements are shown as mean ± SEM of two experiments carried out in triplicates. **D.** Representative Western blot of R1, γH2AX and β-actin (loading control) in CD4^+^ T cells activated for 3 days in the presence of the indicated concentrations of BSO and Au.

A key molecule in DNA synthesis is RNR that converts NDP into dNDP. First, we determined the expression of the regulatory subunit of RNR (R1) in human primary T cells compared to Jurkat T cells. We stimulated naïve CD4^+^ T cells for 72 hours with anti-CD3/CD28 beads and determined R1 expression by Western blot analysis. R1 is only expressed in very low amounts in naïve CD4^+^ T cells. However, upon stimulation R1 is upregulated and within 48 hours the expression level in primary T cells is comparable to that seen in Jurkat T cells (Figure 4A). In the conversion of NDP to dNDP, RNR is oxidized and its further function is dependent on reduction by external electron donors such as Grx/GSH or Trx [21]. If RNR is not reduced, dNDP and thereby dNTP production halts. Therefore dNTP levels can be used as an output for RNR activity [34]. To obtain a sufficient number of cells, we used Jurkat T cells for these analyses. Since dNTP levels fluctuate during the different stages of the cell cycle, we initially synchronized the cells by serum starvation. Upon release from serum starvation, we either left the synchronized cells untreated or treated them with BSO or Au alone or with a combination of both that resulted in a 50% reduction of cells in the S phase as detemined by BrdU staining (Figure 4B). After 16 hours of incubation when the cells had entered S phase, we extracted dNTP and measured the dCTP concentrations as a representative for dNTP. Treatment with either BSO or Au alone did not significantly affect the amount of dCTP synthesized. However, the combination of BSO and Au significantly reduced the amount of dCTP produced (Figure 4C). This indicated that the RNR activity is significantly impaired in T cells when both GSH and Trx are inhibited. Alternatively, inhibition of GSH and Trx might affect the RNR levels in the cells. To rule out this possibility, we determined R1 expression in T cells activated in the presence of different combinations of BSO and Au concentrations. The combination of low concentrations of BSO and Au, which completely abolished DNA synthesis (Figure 3B and 3D), did not affect the expression of R1 (Figure 4D, top row). Taken together, these experiments indicated that the inability of T cells to synthesize DNA when GSH and Trx are inhibited is not caused by a reduced level of RNR but rather by impaired activity of RNR.

If RNR activity and thereby dNTP production are impaired in cells with reduced GSH and Trx, DNA double-stranded breaks (DSB) due to DNA replication stress would be expected in these cells [35, 36]. H2AX, a member of the histone family, is rapidly phosphorylated at serine 139 in response to DSB, and this serine 139-phosphorylated form of H2AX is denoted γH2AX [37, 38]. In order to study whether the cells showed signs of DSB in response to inhibition of GSH and Trx, we stimulated purified CD4^+^ T cells in a checkerboard titration of BSO and Au for 48 h. The cells were subsequently lyzed and their levels of γH2AX determined. Whereas γH2AX was not increased in cells treated with either BSO or Au alone compared to untreated cells, the γH2AX levels were clearly increased in cells treated with the combination of BSO and Au (Figure 4D, middle row). These observations indicated that DNA replication is impaired in cells with reduced GSH and Trx activity and supported that GSH and/or Trx is required for RNR activity and thereby dNTP production.

## DISCUSSION

In this study, we show that exogenous Cys_2_ is indispensable for proliferation of activated human T cells because it is required for production of GSH that subsequently is required for optimal RNR activity and thereby dNDP and dNTP production. For several years, the general paradigm was that T cells cannot import Cys_2_. This paradigm built on early measurements of Cys and Cys_2_ uptake, which indicated that lymphocytes express the ASCT1 and/or ASCT2 transporters for Cys but not the x_c_^−^ transporter for Cys_2_ [7, 9, 39]. Based on these observations it was suggested that a sufficient high concentration of exogenous Cys in the microenvironment is provided to T cells by activated antigen presenting cells [40, 41]. Later it was found that although naïve T cells do not express x_c_^−^, T cell activation strongly induces the expression of x_c_^−^, and it was demonstrated that x_c_^−^ can provide activated T cells with the required amount of Cys_2_ needed for T cell proliferation [1, 8, 10]. One remaining question was why T cell proliferation is dependent on exogenous Cys_2_. Cys together with glutamate and glycine constitute the building blocks of GSH, and it has therefore been suggested that T cells require exogenous Cys_2_/Cys to generate GSH [42, 43]. As GSH is required for optimal T cell proliferation [2, 26–29], a dependency of exogenous Cys_2_ for GSH synthesis would explain the central role of exogenous Cys_2_ in T cell proliferation. However, it has been proposed that T cells can provide Cys for GSH synthesis by an alternative route via the transsulfuration pathway [8] in which methionine is converted to Cys inside the cell completely independently of exogenous Cys_2_. Whether a correlation between the levels of exogenous Cys_2_ and intracellular GSH exists in activated T cells has to our knowledge not been published previously. Here we show that a direct correlation exists between the levels of exogenous Cys_2_ and intracellular GSH in activated human T cells, and that this correlation also encompasses DNA synthesis. Furthermore, T cells activated in tissue culture medium containing Cys_2_ concentrations below 3 μM were completely depleted of GSH. Thus, although the transsulfuration pathway has been described in T cells, this pathway clearly cannot supply T cells with sufficient amounts of Cys for GSH production as recently described for hepatocytes [44]. That transsulfuration cannot supply human T cells for the amount of Cys required for GSH and DNA synthesis was further supported by the observation that blocking Cys_2_ uptake completely inhibited GSH production and DNA synthesis although the cells were cultured in medium containing methionine. In accordance with previous studies [26–28], we found that BSO inhibited GSH production and DNA synthesis in parallel. Thus, when GSH synthesis was blocked, DNA synthesis was halted although Cys_2_/Cys was freely available for the T cells. From these results it could be concluded that in order to proliferate, activated T cells need exogenous Cys_2_ to produce GSH that subsequently is required for optimal DNA synthesis. This is in good agreement with previous studies which found that proliferation and survival of human natural killer cells and primary and malignant B cells are dependent on exogenous Cys_2_/Cys [45–48].

DNA synthesis is, among other factors, dependent on a balanced presence of dNTP generated from RNR-produced dNDP [49, 50]. RNR activity requires an electron donor, which in bacteria, yeast and plants is usually either Trx or Grx [12]. From test tubes experiments using recombinant mouse RNR, it has been shown that both Trx and Grx can reduce mammalian RNR, and that the Grx function is dependent on GSH [21]. We found that GSH was required for optimal DNA synthesis and T cell proliferation; however, we noted some residual DNA synthesis and proliferation in T cells completely depleted of GSH, indicating that other electron donors might be able to substitute for the Grx/GSH pathway. We found a high constitutive expression of Trx in the human leukemic T cell line Jurkat. Combined with the observation that DNA synthesis in Jurkat cells is much more resistant to BSO treatment than DNA synthesis in primary T cells, although the GSH production is equally sensitive to BSO, this supported that Trx can substitute for GSH as electron donor for RNR in human T cells. The synergistic effect of BSO and Au on dNTP production and phosphorylation of H2AX further supported that both GSH and Trx play a role in DNA synthesis. Thus, our study extended previous experiments using recombinant mouse RNR and indicated that Grx/GSH is the primary electron donor for RNR in intact T cells, but that Trx also plays a role and can substitute for Grx/GSH in this process. That either Grx/GSH or Trx can act as electron donor for RNR in intact mammalian cells is supported by the observation that hepatocyte proliferation *in vivo* requires either GSH or at least one functional allele of the TrxR 1 gene [51].

The significance of plasma Cys_2_ and intracellular GSH levels for CD4^+^ T cells in connection with diseases has primarily been studied in relation to HIV. Several studies have shown that HIV infected persons tend to have lower plasma Cys_2_ and intracellular GSH levels compared to healthy controls [52]. In light of this, supplementation with the drug N-acetyl-cysteine (NAC), which increases plasma Cys levels, has been studied in HIV infected persons. Such trials showed that oral supplementation with NAC replenished GSH in lymphocytes and improved the overall T cell function [53]. The importance of Cys_2_ and GSH in relation to HIV infection is underscored by the fact that the decrease in CD4^+^ T cell numbers in the late asymptomatic stage of HIV seemed to coincide with a decrease in plasma Cys_2_ [4], and likewise the concentrations of glutathione reductase was found to be lower in patients with symptomatic HIV than in asymptomatic HIV patients [54]. Whether HIV causes lowered glutathione reductase and GSH levels in CD4^+^ T cells or the lowered levels allow for HIV replication is not known. An increased knowledge of the roles of antioxidants in CD4^+^ T cell biology could help to understand the connection between decreased antioxidant levels and exacerbation of HIV. Interestingly, studies have shown that both BSO and Au could potentially be used in treatment of HIV. BSO in combination with an HDAC inhibitor was shown to be useful in killing latently infected CD4^+^ T cells, as BSO made the infected cells more susceptible to treatment with the HDAC inhibitor. This allowed the use of both drugs at concentrations that were non-toxic for uninfected cells [55]. Au was shown to be more effective in inducing apoptosis in memory CD4^+^ T cells than in naïve CD4^+^ T cells [56]. As memory CD4^+^ T cells are a major reservoir for HIV, Au, like BSO, might be effective in killing latently infected cells when used in combination with other drugs.

In conclusion, in this study we show that exogenous Cys_2_ is required for proliferation of activated human T cells, because Cys_2_ is required for GSH production and GSH is essential for optimal RNR activity and thereby for dNTP synthesis and DNA replication.

## MATERIALS AND METHODS

### Chemicals

L-cystine dihydrochloride (Cys_2_) (C6727), L-buthionine-sulfoximine (BSO) (B2515), 2-mercaptoethanol (2-ME) (M3148) and L-methionine (M5308) were from Sigma-Aldrich Denmark ApS, Brondby, Denmark. Auranofin (EI-206) was from Enzo Life Sciences, Aarhus, Denmark.

### Primary cells

Buffy coats were obtained from anonymous healthy blood donors. Written informed consent was obtained from blood donors at the Department of Clinical Immunology, University Hospital Rigshospitalet, Copenhagen and used without the possibility to identify case specific information. Use of these buffy coats for research was approved by the ethical committee, Region H, The Capital Region of Denmark. Mononuclear cells were isolated from the buffy coats by Lymphoprep™ (Axis-Shield, Oslo, Norway) density gradient centrifugation. Naïve CD4^+^ T cells were subsequently isolated from the mononuclear cell preparation by negative selection using the naïve CD4^+^ T cell isolation kit II (130-094-131, Miltenyi Biotec GmbH, Bergisch Gladbach, Germany) and MACS® Separation Columns (130-042-401, Miltenyi Biotec GmbH) according to the manufacturer's instructions. The purified T cells were cultured in either Dulbecco's Modified Eagle Medium (DMEM, 21013, Invitrogen, Paisly, UK) or X-VIVO 15 medium (1041, Lonza, Verviers, Belgium). DMEM 21013 contains no Cys_2_, methionine, glutamine, glutamic acid or GSH. It was supplemented to a final concentration of 10% fetal bovine serum (FBS), 2 mM glutamine, 0.5 IU/l penicillin, 500 mg/l streptomycin, 50 μM methionine and various concentrations of Cys_2_. X-VIVO 15 medium contains 290 μM Cys_2_, 200 μM methionine, 4 mM glutamine and no GSH and with L-glutamic acid concentrations only known by the company. The cells were stimulated with Dynabeads Human T-Activator CD3/CD28 (111.31D, Invitrogen) at 37°C in 5% CO_2_ at a cell concentration of 0.5 × 10^6^ cells/ml at a bead to cell ratio of two to five.

### Jurkat cells

Jurkat cells (E6) were cultured in RPMI 1640 medium (R5886, Sigma-Aldrich). RPMI 1640 contains 208 μM Cys_2_, 100 μM methionine, 118 μM glutamic acid, 3.25 μM GSH and was supplemented to a final concentration of 10% FBS, 2 mM glutamine, 0.5 IU/l penicillin and 500 mg/l streptomycin. Cells were incubated at 37°C in 5% CO_2_. For cell cycle synchronization, cells were cultured in serum-free RPMI 1640 medium for 48 hours.

### GSH and thioredoxin reductase assays

Measurements of intracellular GSH levels were performed using the Promega GSH-Glo™ Glutathione Assay (V6911, Promega Biotech AB, Nacka, Sweden). This is a luminescence-based assay in which a luciferin derivative is converted into luciferin in the presence of GSH in a reaction that is catalyzed by glutathione S-transferase. The amount of light generated in the firefly luciferase-coupled reaction is proportional to the amount of GSH present in the sample. The assay was performed according to the manufacturer's instructions. We measured only GSH (also called reduced glutathione) and did not include measurements of the oxidized form of glutathione (GSSG). In brief, at the indicated times, the cells were resuspended in 50 μl PBS and transferred to Nunc-Immuno™ MicroWell™ 96 well polystyrene plates. 50 μl GSH-Glo™ Reagent 2X was added to each well, and the plate was incubated for 30 min at room temperature under gentle shaking. 100 μl Luciferin Detection Reagent was added and the luminescence was subsequently measured on a Wallac 1420 Victor^2TM^ Workstation from PerkinElmer.

Measurements of thioredoxin reductase (TrxR) activity were performed using the Abcam® Thioredoxin Reductase (TrxR) Assay Kit (ab83463, Abcam, Cambridge, United Kingdom). This is a colorimetric assay in which reduction of 5,5′-dithiobis (2-nitrobenzoic) acid (DTNB) to 5-thio-2-nitrobenzoic acid (TNB) by TrxR generates a yellow color (λ_max_ 412 nm). The assay was performed according to the manufacturer's instructions. In brief, cells were lyzed in 100 ul assay buffer + Protease/Phosphatase Inhibitor Cocktail (5872S, Cell Signaling) and 12.5 μl lysate was used for each reaction. As other enzymes, such as glutathione reductase and glutathione peroxidase, can also reduce DTNB, two measurements were performed for each sample; the first measures total DTNB reduction in the sample, and the second measures DTNB reduction in the presence of a TrxR specific inhibitor. The absorbance was measured at 405 nm several times for up to 45 minutes following setup of the assay. A TNB standard curve and a TrxR positive control were included for each assay. Enzyme activity in each sample was calculated from the standard curve.

### Western blot analysis

Western blot analysis was carried out as previously described [57, 58]. In short, cells were stimulated with Dynabeads Human T-Activator CD3/CD28 for the time indicated at 37°C, lysed in 1% Triton X-100 lysis buffer + Protease/Phosphatase Inhibitor Cocktail (5872S, Cell Signaling) and run on 10% polyacrylamide gels. The proteins were transferred to Amersham Hybond ECL nitrocellulose sheets (RPN2020D, GE Healthcare, Brondby, Denmark) and visualized using ECL technology (RPN2232, GE Healthcare). As primary antibodies we used rabbit polyclonal anti-H2AX phospho S139 (1:10000) (ab11174, Abcam, Cambirdge, UK), rabbit polyclonal anti-GAPDH (1:5000) (ab9485, Abcam), rabbit monoclonal anti-Trx (1:1000) (2429, Cell Signaling, (via BioNordika Denmark A/S, Herlev, Denmark)), rabbit polyclonal anti-R1 (1:1000) (3388, Cell Signaling), and mouse monoclonal anti-β-actin (1:1000) (3700S, Cell Signaling). Secondary antibodies included HRP-conjugated polyclonal swine anti-rabbit Ig (1:1000) (P0399, DAKO, Glostrup, Denmark) and HRP-conjugated polyclonal rabbit anti-mouse Ig (1:2000) (P0260, DAKO).

### DNA synthesis

DNA synthesis was measured by ^3^H-thymidine and 5-bromo-2′-deoxyuridine (BrdU) incorporation. The ^3^H-thymidine incorporation assay was carried out as previously described [59]. Briefly, cells were incubated for 48 or 72 hours at 37°C in 5% CO_2_, and 20 μl medium (X-VIVO, D-MEM or RPMI-1640) containing 50 μCi/ml [Methyl-^3^H]-thymidine (NET027005MC, Perkin Elmer) was subsequently added to each well resulting in a specific activity of 1 μCi/well. The incubation was continued for another 6 hours before the cells were harvested and ^3^H-thymidine incorporation was quantified as counts per minute (cpm) using a scintillation counter. BrdU incorporation assays were performed using the FITC BrdU Flow Kit (BD Biosciences, San Diego, CA, USA) according to supplier's protocol and as previously described [60]. In short, cultured cells were incubated with 17.7 μM BrdU for 1 hour and subsequently fixed, permeabilized and treated with DNase. Afterwards the cells were stained with FITC anti-BrdU and 7-AAD and analyzed by flow cytometry on a FACS Calibur.

### Cell division assay

CFSE (C34554, Life Technologies, Naerum, Denmark) was used to stain cells and measure cell division as previously described [61, 62]. In short, purified naïve CD4^+^ T cells were resuspended in 4 ml 37°C PBS. CFSE was added to the cells at a final concentration of 2.5 μM CFSE. The cells were then incubated for 9 minutes at 37°C. FBS was added to terminate the incorporation of CFSE. Following CFSE loading, the cells were incubated in X-VIVO medium at 37°C in 5% CO_2_ with anti-CD3/CD28 beads and the indicated inhibitors. After 72 hours, the cells were analyzed by flow cytometry on a FACS Calibur.

### dNTP extraction and quantification

dNTP extraction and quantification was performed as previously described [63]. For dNTP extraction, cells were washed in Hanks Balanced Salt solution and resuspended in 60% ice-cold methanol before being lysed using ultrasound. The cell debris was pelleted by centrifugation at 16.000 g for 15 min, 4°C. Supernatants were transferred to Amicon Ultra centrifugal filters (UFC500324, Merck Milipore, Hellerup, Denmark) and centrifuged at 14.000 g for 30 min, 4°C to remove macromolecules larger than 3 kDa. Liquid was removed from the sample by freeze drying, and the resultant pellet was resuspended in 12 μl nuclease-free H_2_O. The samples were either used directly for the assay or stored at −80°C. For dNTP quantification, 5 μl of each sample was mixed with 20 μl mastermix (1 μl nucleotide detection primer (10 μM), 1 μl dCTP detection template (10 μM), 1 μl of FAM-dCTP probe (10 μM), 2 μl of MgCl2 (25 mM), 1 μl of dNTPs (2.5 mM), 2.5 μl of 10X PCR Buffer II, 0.175 μl of AmpliTaq Gold Polymerase (5U/μl) (4311816, Applied Biosystems, Life Technologies), and 11.325 μl of nuclease-free H2O). The qPCR program included a 10 minute hot start at 95°C followed by 75 minutes at 60°C, while measuring the fluorescence intensity every 1 minute. The raw fluorescence spectra for each well were exported to excel and analyzed. A dCTP standard curve was included for each assay using dCTP from Life Technologies (10297–018). The following primers were used: Nucleotide detection primer 5′-CCG CCT CCA CCG CC-3′, dCTP detection template 5′-CCA CTC ACT CTT ACC TCA ATC CTT TGT TTG GCG GTG GAG GCG G-3′, and FAM-dCTP probe 5′-/6FAM/AGG ATT GAG/ZEN/GTA AGA GTG AGT GG/IABkFQ/-3′. 6FAM (6-carboxyfluorescein), ZEN (non-abbreviation), IABkFQ (Iowa black fluorescein quencher).

### Statistical analysis

Data is shown as mean ± SEM, significance is calculated by using unpaired Student's *t*-test. * indicates *p* ≤ 0.05, **p ≤ 0.01 and ***p ≤ 0.001.
